# Expression of a Dominant Negative CELF Protein *In Vivo* Leads to Altered Muscle Organization, Fiber Size, and Subtype

**DOI:** 10.1371/journal.pone.0019274

**Published:** 2011-04-26

**Authors:** Dara S. Berger, Michelle Moyer, Gregory M. Kliment, Erik van Lunteren, Andrea N. Ladd

**Affiliations:** 1 Department of Cell Biology, Lerner Research Institute, Cleveland Clinic, Cleveland, Ohio, United States of America; 2 Cleveland Veterans Affairs Medical Center, Cleveland, Ohio, United States of America; 3 Pulmonary and Critical Care Division, Department of Medicine, Case Western Reserve University, Cleveland, Ohio, United States of America; Centre de Regulació Genòmica, Spain

## Abstract

**Background:**

CUG-BP and ETR-3-like factor (CELF) proteins regulate tissue- and developmental stage-specific alternative splicing in striated muscle. We previously demonstrated that heart muscle-specific expression of a nuclear dominant negative CELF protein in transgenic mice (MHC-CELFΔ) effectively disrupts endogenous CELF activity in the heart *in vivo*, resulting in impaired cardiac function. In this study, transgenic mice that express the dominant negative protein under a skeletal muscle-specific promoter (Myo-CELFΔ) were generated to investigate the role of CELF-mediated alternative splicing programs in normal skeletal muscle.

**Methodology/Principal Findings:**

Myo-CELFΔ mice exhibit modest changes in CELF-mediated alternative splicing in skeletal muscle, accompanied by a reduction of endomysial and perimysial spaces, an increase in fiber size variability, and an increase in slow twitch muscle fibers. Weight gain and mean body weight, total number of muscle fibers, and overall muscle strength were not affected.

**Conclusions/Significance:**

Although these findings demonstrate that CELF activity contributes to the normal alternative splicing of a subset of muscle transcripts *in vivo*, the mildness of the effects in Myo-CELFΔ muscles compared to those in MHC-CELFΔ hearts suggests CELF activity may be less determinative for alternative splicing in skeletal muscle than in heart muscle. Nonetheless, even these small changes in CELF-mediated splicing regulation were sufficient to alter muscle organization and muscle fiber properties affected in myotonic dystrophy. This lends further evidence to the hypothesis that dysregulation of CELF-mediated alternative splicing programs may be responsible for the disruption of these properties during muscle pathogenesis.

## Introduction

Pre-mRNA alternative splicing is a common mechanism for generating transcript and protein diversity. It is estimated that more than 90% of human genes produce alternatively spliced transcripts, with the majority of these exhibiting tissue-specific regulation [1], [2]. Two members of the CUG-BP and ETR-3-like factor (CELF) family, CELF1 (also known as CUG-BP1) and CELF2 (also known as CUG-BP2 or ETR-3), are expressed in heart and skeletal muscle, where they have been shown to be key regulators of tissue- and developmental stage-specific alternative splicing [3]–[6]. CELF1 and CELF2 protein levels are normally down-regulated in vertebrate heart and skeletal muscle shortly after birth [4], [6]. This regulation occurs post-transcriptionally, involving destabilizing modifications of the CELF1 protein and microRNA-mediated repression of translation of CELF1 and CELF2 transcripts [7], [8]. In the heart, a recent study found that nearly half of transcripts that undergo fetal-to-adult alternative splicing transitions in mice respond to over-expression of CELF1, suggesting that the level of CELF activity is determinative for alternative splicing of these transcripts in heart [4]. CELF proteins have also been implicated in regulating fetal versus adult splicing patterns of some skeletal muscle transcripts [9], [10].

The postnatal down-regulation of CELF proteins is critical, as too much CELF activity in mature heart and skeletal muscle is pathogenic. In patients with myotonic dystrophy type 1 (DM1), an adult-onset form of muscular dystrophy caused by the expression of mutant transcripts from the *DMPK* gene containing expanded CUG repeats [11]–[13], CELF1 is aberrantly up-regulated in the heart and skeletal muscles [10], [14]. CELF targets in these tissues exhibit fetal splicing patterns, and developmental stage-inappropriate transcripts have been directly implicated in disease symptoms [9], [10], [15]. CELF1 protein levels are also up-regulated in heart and skeletal muscle in transgenic mice following induction of expanded CUG repeat RNA expression [16], [17]. The expression of expanded CUG repeat RNAs or over-expression of CELF1 in heart and skeletal muscle recapitulate many of the aberrant alternative splicing patterns observed in DM1 patient tissues, as well as other features of DM1 pathogenesis [16]–[20].

Too little CELF activity can also be pathogenic. We previously demonstrated that targeted repression of CELF activity in the postnatal mouse heart by expression of a nuclear dominant negative CELF protein (NLSCELFΔ) under the control of the cardiac muscle-specific α-myosin heavy chain promoter (MHC-CELFΔ) leads to not only changes in alternative splicing of target transcripts, but also cardiac hypertrophy, chamber dilation, severe cardiac dysfunction, and premature death [21], [22]. These results indicate that the persistence of some CELF activity in postnatal heart muscle is critical for the maintenance of developmental stage-appropriate alternative splicing and healthy cardiac function.

In this study, we use a similar dominant negative approach to explore the role of CELF-mediated alternative splicing regulation in normal skeletal muscle. Although previous studies have shown that over-expression of CELF1 in skeletal muscle is sufficient to disrupt alternative splicing and muscle phenotype [18], [20], this is the first study to test whether CELF activity is normally required in skeletal muscle by specifically repressing CELF activity. The dominant negative approach has several advantages over a gene knockout approach. First, dominant negative expression can be easily targeted to a specific tissue using a tissue-specific promoter without the need to use a bitransgenic Cre-lox system. Second, the dominant negative protein will simultaneously inhibit the activities of all CELF proteins in that tissue, in this case CELF1 and CELF2. And third, the dominant negative protein contains a strong nuclear localization signal, so its repression should be limited to the nucleus where alternative splicing takes place, leaving any cytoplasmic functions of the CELF proteins unperturbed. Here we report that skeletal muscle-specific expression of NLSCELFΔ under control of the myogenin promoter (Myo-CELFΔ) leads to modest changes in alternative splicing in the muscles of some, but not all, CELF targets previously identified in the heart. These splicing changes are accompanied by changes in endomysial and perimysial spacing, increased variation in muscle fiber size, and switching of muscle fiber subtypes. Body weight, fiber number, and overall muscle strength were not impaired. The mildness of the Myo-CELFΔ skeletal muscle phenotype is in stark contrast to the strong phenotype observed in the hearts of MHC-CELFΔ mice, in which both alternative splicing and cardiac function are significantly disrupted by expression of NLSCELFΔ [21], [22]. These data suggest that while CELF-mediated alternative splicing regulatory programs participate in the establishment and/or maintenance of skeletal muscle organization and fiber properties, CELF activity may be less determinative for alternative splicing in skeletal muscle than in heart.

## Results

### Transgenic lines of Myo-CELFΔ mice were generated that express different levels of dominant negative protein

**Figure 1:**
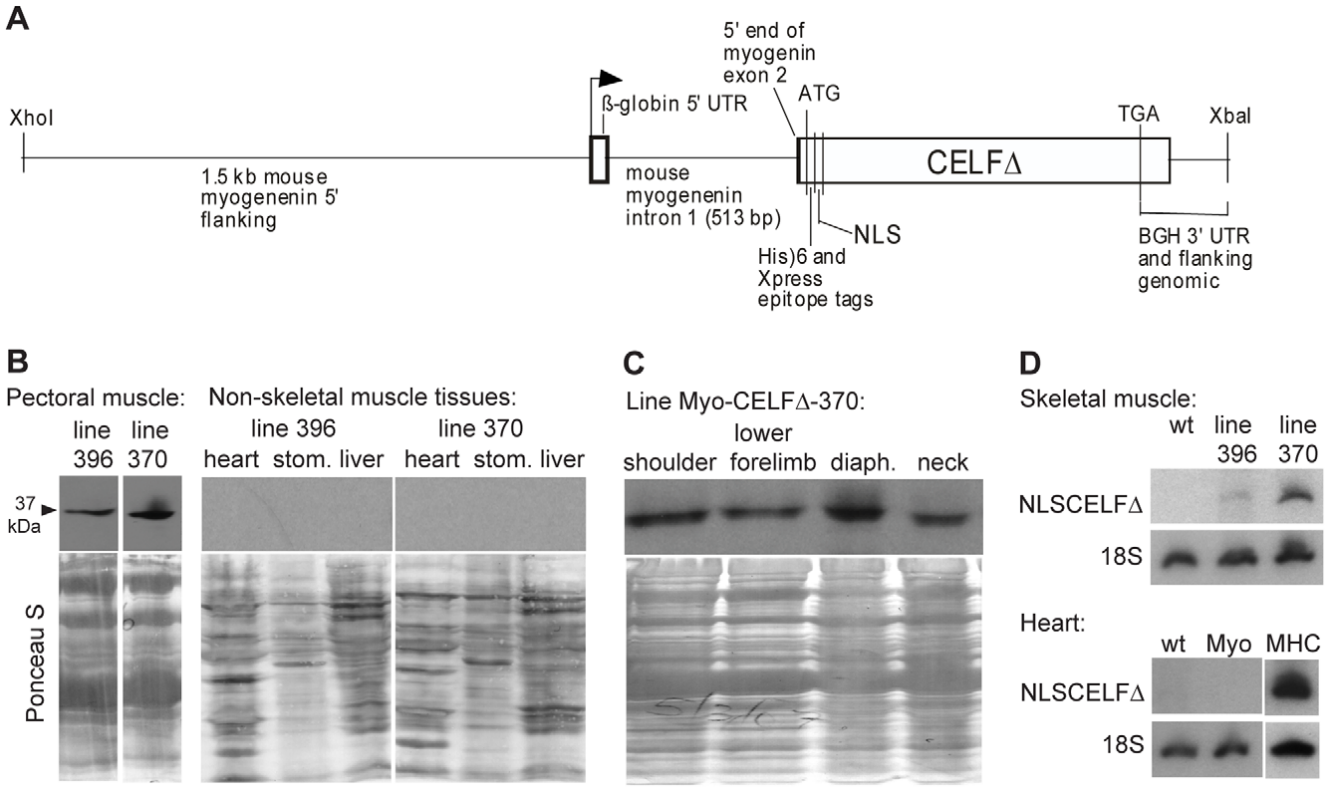
Myo-CELFΔ transgenic mice express the nuclear dominant negative CELF protein in skeletal muscles. (**A**) The Myo-CELFΔ transgene contains NLSCELFΔ behind the mouse myogenin promoter. (**B**) Expression of NLSCELFΔ protein in skeletal muscle, but not heart, stomach (stom.), or liver, was confirmed in total protein lysates from tissues of Myo-CELFΔ mice by western blot using an antibody against the N-terminal Xpress epitope tag. Equivalent loading was confirmed by Ponceau S staining. Intervening lanes with samples from non-expressing lines were excised from the skeletal muscle blot. (**C**) Expression of NLSCELFΔ protein in a variety of Myo-CELFΔ skeletal muscles was confirmed by western blot using an antibody against the N-terminal Xpress epitope tag. Protein loading and integrity was confirmed by Ponceau S staining. (**D**) Expression levels of NLSCELFΔ were compared between Myo-CELFΔ lines by semi-quantitative RT-PCR. Lack of NLSCELFΔ transcripts in the Myo-CELFΔ heart (Myo) was confirmed; RNA from the heart of an MHC-CELFΔ-10 mouse (MHC), in which NLSCELFΔ is highly expressed in cardiac muscle, was used as a positive control.

A truncated CELF protein, CELFΔ, exhibits dominant negative activity as defined by several criteria: (1) alone it has no effect on alternative splicing when expressed in cells lacking detectable levels of endogenous CELF proteins, (2) it inhibits changes in alternative splicing induced by wild type CELF proteins when co-expressed, and (3) it has no effect on non-CELF mediated alternative splicing [3]. A modified form of this protein, NLSCELFΔ, retains its dominant negative activity and is restricted to the nucleus [21]. Heart muscle-specific expression of NLSCELFΔ in MHC-CELFΔ mice has been shown to specifically disrupt cardiac CELF-mediated alternative splicing *in vivo* and leads to cardiomyopathy, severe cardiac dysfunction, and premature death [21], [22]. Cardiac hypertrophy, survival, and alternative splicing of CELF targets were all rescued by crossing MHC-CELFΔ mice to mice over-expressing CELF1 in the heart, indicating that both the molecular and gross phenotypes can be attributed to loss of CELF activity [21]. To create mice that express this nuclear dominant negative protein specifically in skeletal muscle, a transgene was created (Myo-CELFΔ; Figure 1A) in which expression of NLSCELFΔ is driven by the mouse myogenin promoter. The myogenin promoter was chosen because it drives expression specifically in cells of the skeletal muscle lineage, beginning prior to terminal differentiation and continuing into adulthood [23].

Several transgenic founders with germ line transmission were obtained. First generation offspring were screened for expression of the NLSCELFΔ protein in skeletal muscle by western blotting, and three lines (Myo-CELFΔ-360, -370, and –396) with different levels of detectable transgene expression were obtained. Transgenic animals from the lowest-expressing line, Myo-CELFΔ-360, became severely obese at a young age. As obesity was not observed in any other line despite much higher levels of NLSCELFΔ expression, this can likely be attributed to the integration site of the transgene. Given the confounding effects that severe obesity could have on muscle health and function, the Myo-CELFΔ-360 line was culled without further analysis. Thus, only the highest- and mid-expressing lines, Myo-CELFΔ-370 and -396, respectively (Figure 1B), were selected for further study.

The initial screen for transgene expression was conducted on pectoral muscle samples. The myogenin promoter should drive transgene expression in late myogenic precursors and differentiated skeletal muscles throughout the body, but not in non-skeletal muscle tissues [23], [24]. To confirm this expression pattern for the Myo-CELFΔ transgene, several other tissues were examined by western blot. NLSCELFΔ protein expression was not detected in the heart (cardiac muscle), stomach (smooth muscle), or liver (non-muscle) tissues of Myo-CELFΔ mice (Figure 1B). NLSCELFΔ protein expression was strongly detected in shoulder (trapezius), lower forelimb (extensor), diaphragm, and neck muscles in both Myo-CELFΔ-370 and -396 lines (Figure 1C and data not shown). To quantify the difference in expression levels between Myo-CELFΔ lines, semi-quantitative RT-PCR was performed on RNAs from these same skeletal muscle tissues (Figure 1D). While no NLSCELFΔ transcripts were detected in wild type skeletal muscles, NLSCELFΔ transcripts were detected in all skeletal muscles evaluated for both Myo-CELFΔ lines, with an average of 7.9±1.7 fold higher expression in Myo-CELFΔ-370 compared to Myo-CELFΔ-396 muscles. No NLSCELFΔ transcripts were detected in Myo-CELFΔ hearts.

### CELF-mediated alternative splicing is modestly disrupted in Myo-CELFΔ muscle

**Figure 2:**
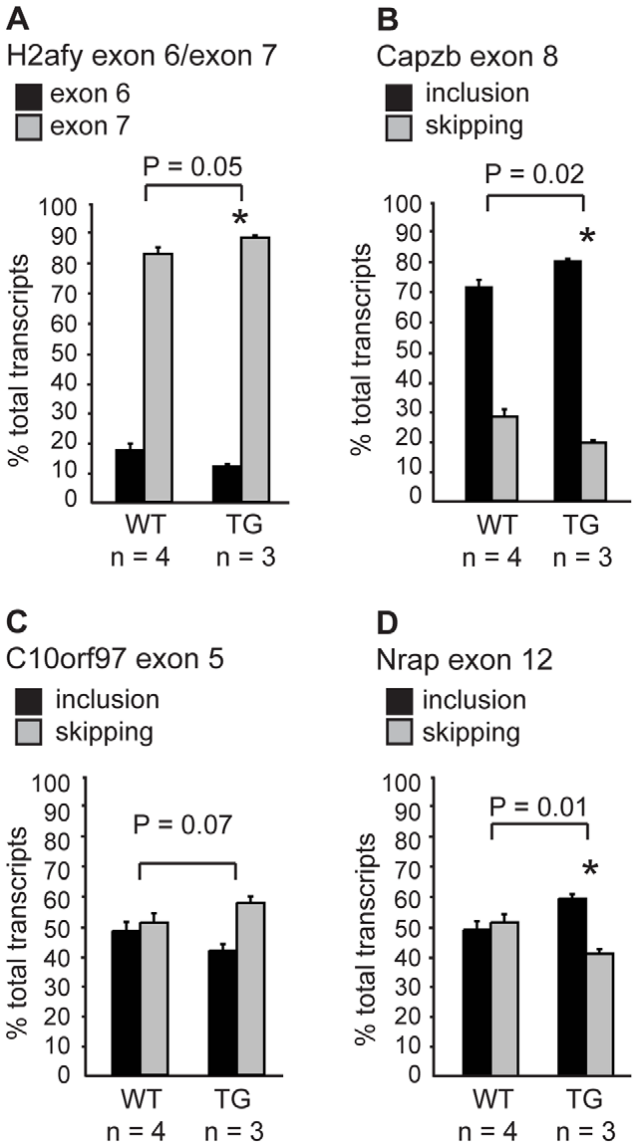
The alternative splicing of some transcripts is affected in Myo-CELFΔ muscle. The alternative splicing of transcripts that were previously identified as targets of CELF regulation in the mouse heart was evaluated in the pectoral muscles of nonparous female wild type and Myo-CELFΔ-370 siblings at 24 weeks. H2afy mutually exclusive exons 6 and 7 (**A**) and Capzb exon 8 (**B**) exhibited significant changes in splicing. (**C**) C10orf97 exon 5 showed a trend towards decreased inclusion in pectoral muscle. Although not statistically significant in pectoral muscle, a significant decrease in exon inclusion was observed in thigh muscle (data not shown; see text). (**D**) Nrap exon 12, which is repressed in DM1 skeletal muscle, displayed a significant increase in inclusion in Myo-CELFΔ muscle. An asterisk denotes a significant difference from wild type (P≤0.05).

The majority of CELF targets identified to date were found in the developing heart [4], [21]. Few CELF targets have been identified and characterized in skeletal muscle. Alternative exons 6b and 7a of the skeletal muscle-specific chloride channel ClC-1 are aberrantly included in DM1 skeletal muscle where CELF1 levels are elevated [9], but are 100% skipped in normal muscle and do not respond to further loss of CELF activity in Myo-CELFΔ mice (data not shown). Therefore, to confirm that the alternative splicing activity of the CELF family is disrupted in Myo-CELFΔ mice, alternative splicing patterns of transcripts previously identified as targets of CELF regulation in the heart [4], [21] were compared in pectoral muscles from sex- and age-matched wild type and transgenic mice (Figure 2 and Table 1). Of the twelve known targets tested, two demonstrated significant differences in exon inclusion between wild type and transgenic siblings: H2afy mutually exclusive exons 6 and 7 and Capzb exon 8 (Figure 2A, B). In both cases, the direction of change that was observed was opposite to that seen in the hearts of mice over-expressing CELF1 [4], which is consistent with a loss of CELF activity in Myo-CELFΔ muscle. There was also a trend toward decreased inclusion of C10orf97 exon 5 in Myo-CELFΔ pectoral muscle (Figure 2C), which is likewise opposite of the pattern seen in CELF1-over-expressing mice [4]. Although the decrease in inclusion did not quite reach statistical significance for C10orf97 exon 5 in pectoral muscle, a significant decrease was found in thigh muscles from the same mice (41.4±1.8% in wild type versus 32.5±3.7% inclusion in Myo-CELFΔ-370 mice, P = 0.01). Nine other previously identified cardiac targets showed no significant change in alternative splicing in Myo-CELFΔ-370 muscle (Table 1). It should be noted that two of these that were predicted to exhibit increases in exon inclusion based on patterns observed in the heart [4], [21], Mfn2 exon 3 and Bin 1 exon 10, already had baseline levels of inclusion close to 100% allowing no room for an additional shift. In addition to the previously identified CELF targets, we examined the alternative splicing of Nrap exon 12, a transcript identified as a novel putative target in another ongoing study in the lab. Although Nrap exon 12 was not previously described as a target of the CELF proteins, its inclusion is reduced in DM1 muscle [25], which has elevated CELF1 protein levels [10]. We found that Nrap exon 12 inclusion is elevated in Myo-CELFΔ muscle (Figure 2D). Taken together, these data suggest that CELF proteins negatively regulate Nrap exon 12 inclusion.

**Table 1:** Alternative splicing of unaffected CELF targets.

Alternative region	Predicted effect on	% inclusion in pectoral muscle (n) c		P value
	inclusion	Wild type	Myo-CELFΔ	
Ank2 exon 21	a Increase	89.3±0.6 (4)	90.2±1.3 (3)	0.31
Atp2b1 exon 21	a Increase	56.1±3.4 (4)	59.2±4.0 (3)	0.29
Mfn2 exon 3	a Increase	95.1±0.6 (3)	95.3±2.4 (3)	0.46
Ppfibp1 exon 4	a Increase	36.5±2.8 (4)	40.0±4.1 (3)	0.26
Sorbs1 exon 6	a Increase	58.0±1.3 (3)	55.0±1.9 (3)	0.14
Xpo7 exon 4 5′ss	a Increase	50.3±1.4 (4)	51.4±0.9 (3)	0.25
Bin 1 exon 10	b Increase	98.2±0.3 (3)	98.5±0.2 (3)	0.19
Itgb1 exon D	b Decrease	49.7±3.4 (4)	49.7±3.1 (3)	0.50
Mef2A exon 16	b Decrease	85.9±1.5 (4)	88.1±1.7 (3)	0.20

a Based on the effect of over-expressing CELF1 in the heart (i.e. the opposite direction) [4].

b Based on the effect of repressing CELF activity in the heart (i.e. the same direction) [21].

c n =  number of samples in group.

### Myo-CELFΔ transgenic mice exhibit changes in muscle organization and fiber size

Hemizygous transgenic animals from both the higher-expressing Myo-CELFΔ-370 and lower-expressing Myo-CELFΔ-396 lines were obtained at the expected Mendelian frequency, survive to maturity, and live normal life spans. No significant differences in mean body weight were observed for either males or females from the Myo-CELFΔ-370 line when compared to sex-matched wild type littermates over a sixteen-week time course, suggesting that there are no gross changes in overall muscle mass or body morph (Figure S1). Myo-CELFΔ transgenic mice also exhibit no obvious impairment in mobility or gait.

**Figure 3:**
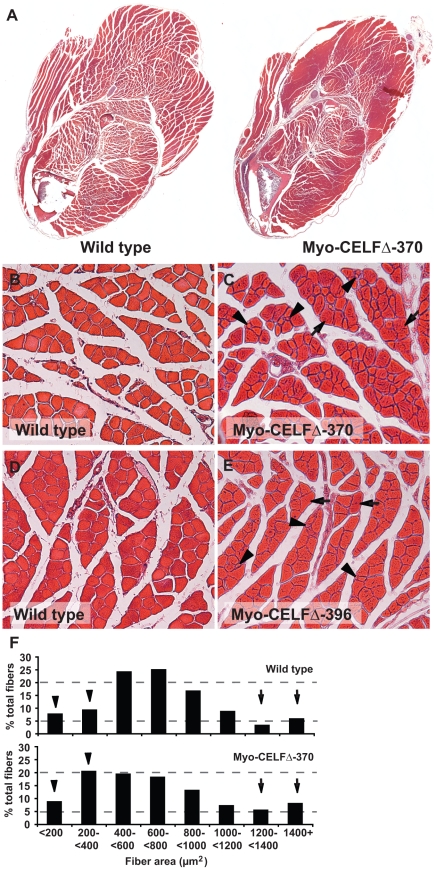
Myo-CELFΔ mice have altered skeletal muscle organization and variable muscle fiber size. (**A**) Transverse cross-sections of the lower forelimb of adult Myo-CELFΔ-370 mice reveal a reduction in the endomysial and perimysial spaces relative to that of a wild type littermate. Sections shown are representative of three independently processed sex- and age-matched littermate pairs. Higher magnification views of muscles from wild type (**B**) and Myo-CELFΔ-370 (**C**) littermates or wild type (**D**) and Myo-CELFΔ-396 (**E**) littermates reveal an increased prevalence of both enlarged (arrows) and diminutive (arrowheads) muscle fibers in transgenic mice. In order to ensure that the same muscle groups are being compared between wild type and transgenic mice, the regions shown in (**B**) and (**C**) are from a portion of the cross-section where the reduction in spacing around the muscle fibers in the Myo-CELFΔ-370 limb was less pronounced. (**F**) Fiber area was measured for muscle fibers within matched regions of hind limb sections from two sex- and age-matched MyoCELFΔ-370 and wild type pairs. Fiber size varied more in transgenic muscles, with an enrichment of both smaller (arrowheads) and larger (arrows) fibers relative to wild type muscles.

To more closely examine the skeletal muscle of Myo-CELFΔ mice, we performed histological analysis on transverse cross-sections of limbs from adult transgenic mice and their wild type littermates. Although there were no apparent deficiencies in the amount or distribution of muscle within the lower hind limb, the endomysial and perimysial spaces were considerably reduced in the Myo-CELFΔ-370 mice (Figure 3A). Similar differences were observed in sections from the forelimb and upper hind limb (data not shown). To quantify the extent of the reduction in interstitial spacing, the interstitial area was measured in four different large muscle groups in sections of lower hind limbs from two age- and sex-matched pairs of Myo-CELFΔ-370 and wild type mice. While nearly one third of the total muscle area was interstitial space in wild type muscles (32.8**±**3.6% of the total area), interstitial spacing was significantly reduced in Myo-CELFΔ-370 muscles (21.6**±**7.4%, P = 0.01). Although there appeared to be a slight reduction in the interstitial spaces in portions of cross-sections from Myo-CELFΔ-396 limbs, the differences were far less pronounced and less consistent than in the higher-expressing Myo-CELFΔ-370 line (data not shown); comparison of matched fields of lower hind limb sections from Myo-CELFΔ-396 transgenic and wild type littermates revealed no significant difference in total interstitial area. It should be noted that lymphatic masses were also observed in some cross-sections from the upper limbs of Myo-CELFΔ-370 but not wild type mice; these are likely to be extensions of the auxiliary lymph nodes. Although this could be indicative of cancerous growth of lymphatic tissues (perhaps due to an integration site effect in this line), there is no discernable effect on the health or lifespan of Myo-CELFΔ-370 mice.

Higher magnification of the limb muscles reveals changes in muscle fiber size in the Myo-CELFΔ mice (Figure 3B-E). Although the muscle fibers within a region are roughly similar in size in wild type mice (Figure 3B, D), muscle fiber size is highly variable in Myo-CELFΔ-370 mice, ranging from very small to very large (Figure 3C). To quantify differences in fiber size, the areas of individual fibers were measured in several fields from matched regions of muscle in hind limb sections from wild type and Myo-CELFΔ-370 mice. Although the average fiber area did not differ between wild type and transgenic mice (719.7**±**10.9 µm^2^, n = 1251 fibers for wild type versus 707.7**±**11.7 µm^2^, n = 1719 fibers for Myo-CELFΔ-370, P = 0.22), the variance, which is a measure of the variability of the data, was significantly different (P<0.0001). Whereas most fibers tend to cluster within a size range close to the mean in wild type mice, fiber size is more broadly distributed in Myo-CELFΔ-370 mice, with a larger percentage of fibers falling at the ends of the spectrum (Figure 3F). Differences were particularly pronounced at the extreme end of the range; the maximum fiber size measured in Myo-CELFΔ-370 muscle was nearly twice that of the maximum fiber size in wild type (4927 µm^2^ versus 2593 µm^2^). Although both diminutive and enlarged fibers are also seen in Myo-CELFΔ-396 muscle (Figure 3E), fiber size is more uniform in the lower-expressing Myo-CELFΔ-396 line than in the Myo-CELFΔ-370 line, and the overall variance in fiber sizes does not differ significantly from that of wild type (P = 0.36). No differences in the number of muscle fibers were detected in Myo-CELFΔ mice (Figure S2).

### Myo-CELFΔ transgenic mice exhibit changes in muscle fiber subtype

**Figure 4:**
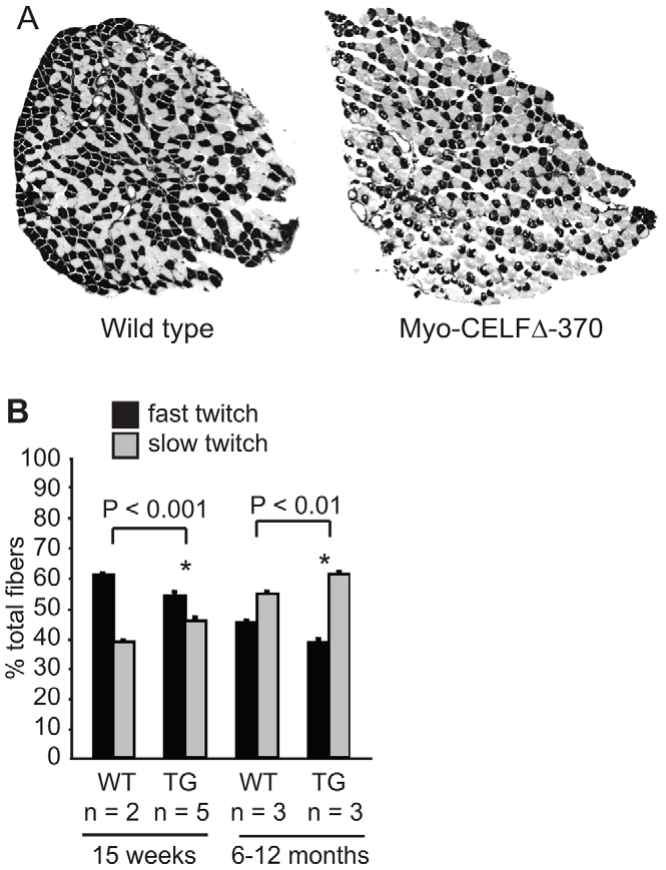
Fiber type composition is altered in Myo-CELFΔ mice. (**A**) Representative sections of soleus muscles isolated from 15 week old wild type and Myo-CELFΔ-370 littermates that have been stained for myofibrillar actomyosin ATPase activity are shown. Pale fibers  =  slow twitch (Type I), dark fibers  =  fast twitch (Type II). (**B**) The number of slow twitch (pale) and fast twitch (dark) fibers were counted in 15 week old or 6–12 month old wild type and Myo-CELFΔ-370 littermates and mean values were calculated for each group. An asterisk denotes a significant difference from wild type (P≤0.05).

Skeletal muscle is composed of two basic fiber types. Slow twitch (Type I) fibers are aerobic, fire slowly, and are resistant to fatigue. Fast twitch (Type II) fibers are often anaerobic, and both fire and fatigue more quickly than slow twitch fibers. To determine whether the fiber type composition is altered in Myo-CELFΔ mice, sections of isolated soleus muscles from sex- and age-matched wild type and Myo-CELFΔ mice were stained for myofibrillar actomyosin ATPase under basic conditions. Using this protocol, the Type I ATPase is alkaline labile, leaving slow twitch fibers pale, while Type II ATPase is alkaline stabile, resulting in dark staining of fast twitch fibers. The soleus muscle was chosen for this analysis because it normally has a mixture of slow and fast twitch fiber types in mice, so a shift towards either subtype would be detectable. A small but significant shift from fast to slow twitch fibers was observed in Myo-CELFΔ-370 mice (Figure 4). Although the baseline percentage of fast twitch fibers changed in wild type mice between 15 weeks and six months (Figure 4B), the percentage of fast twitch fibers in Myo-CELFΔ-370 mice decreased proportionally. A similar shift in the proportion of fast to slow twitch muscle was also observed in a pair of sex- and age-matched adult Myo-CELFΔ-396 littermates (data not shown).

### Muscle performance is not affected in Myo-CELFΔ mice

**Figure 5:**
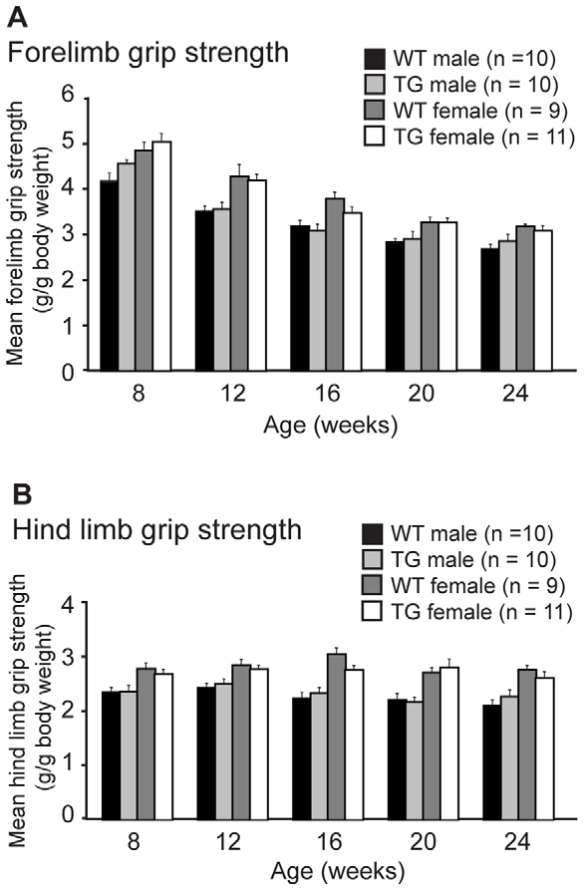
Grip strength is unaffected in Myo-CELFΔ mice. Forelimb (**A**) and hind limb (**B**) grip strengths were measured every four weeks in a cohort of wild type and Myo-CELFΔ-370 siblings over a sixteen week time course. Force measurements were normalized against body weight. Males and females were assessed independently due to sex differences in muscle mass and body weight. No significant differences were found between sex-matched wild type and transgenic mice at any age evaluated.

To assess whether muscle strength is altered in Myo-CELFΔ mice, a longitudinal assessment of *in vivo* grip strength was conducted for wild type and Myo-CELFΔ-370 mice. Fore- and hind limb grip strengths were measured every four weeks over a time course from eight to 24 weeks of age and normalized to body weight. No significant differences were observed between sex- and age-matched wild type and Myo-CELFΔ-370 mice in either fore- or hind limb grip strength at any age tested, indicating that there is no overt loss or gain of limb muscle strength in the transgenic mice (Figure 5).

**Figure 6:**
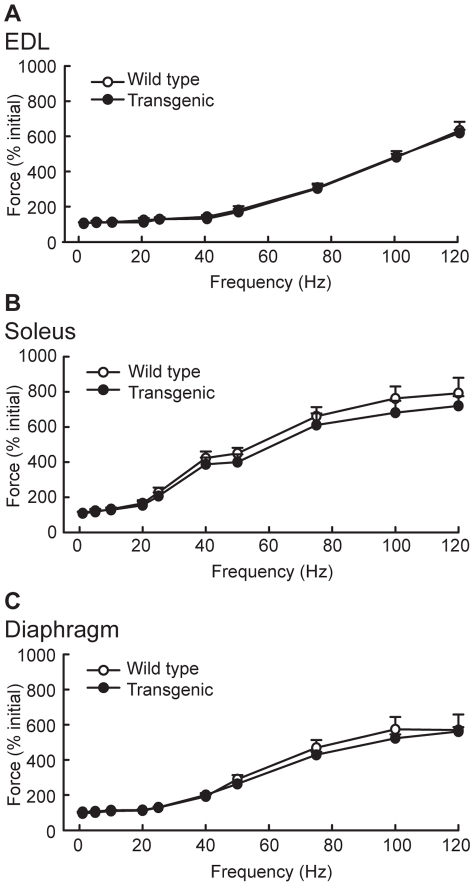
Force measurements are not affected in skeletal muscles from Myo-CELFΔ mice. Force was assessed *ex vivo* in extensor digitorum longus (EDL) (**A**), soleus (**B**), and diaphragm (**C**) muscles isolated from sex- and age-matched wild type and Myo-CELFΔ-370 mice. No significant differences were found at any frequency.

Muscle performance was also assessed *ex vivo*. Force, fatigue, and contraction and relaxation times were compared between isolated extensor digitorum longus (EDL), soleus, and diaphragm muscles from wild type and Myo-CELFΔ-370 mice. Values for force did not differ at any stimulation frequency between wild type and transgenic mice in any of the three muscles evaluated (Figure 6), consistent with these mice exhibiting no differences in overall limb muscle strength *in vivo* (Figure 5). Myo-CELFΔ-370 mice also exhibited no differences in fatigue resistance relative to wild type when any of the muscles were stimulated repetitively with 25 or 50 Hz train stimulation (Figure S3). Contraction time and half relaxation times were calculated as measures of contractile performance. The contraction time did not differ significantly in Myo-CELFΔ-370 mice in any of the muscles evaluated with stimulation at either 25 or 50 Hz (Figure S4). The half relaxation time did not differ at either frequency in EDL or soleus, but was significantly increased at some time points in diaphragm at 50 Hz (Figure S5). Although significant differences were seen only in parts of the stimulation train, an increase in the half relaxation times are consistent with a shift towards more slow twitch fibers. Taken together with the *in vivo* data, these *ex vivo* results indicate that disruption of CELF-mediated alternative splicing in the skeletal muscle by repression of CELF activity neither enhances nor impedes muscle performance.

## Discussion

Only some of the transcripts that were previously identified as being targets of CELF-mediated alternative splicing regulation in the heart [4], [21] showed a demonstrable change in alternative splicing in Myo-CELFΔ muscle (Figure 2 and Table 1). The changes that were observed were generally mild, not exceeding ∼12% change in the level of exon inclusion relative to the total pool of transcripts. The mildness or lack of effect on alternative splicing in Myo-CELFΔ mice may reflect a lesser regulatory role for CELF proteins in skeletal muscle. Alternative splicing outcomes are the result of combinatorial regulation of exon recognition by multiple, sometimes antagonistic, factors that may vary between tissues and developmental stages. CELF activity may therefore be determinative for inclusion of an exon in heart, but not in skeletal muscle. Consistent with this idea, alternative splicing is far less disrupted in skeletal muscle in Myo-CELFΔ mice than in the hearts of MHC-CELFΔ mice. For example, inclusion of alternative exons in Itgb1 and Mef2A transcripts is significantly reduced in the hearts of both MHC-CELFΔ lines [22], but remains unaffected in Myo-CELFΔ-370 pectoral muscles (Table 1). NLSCELFΔ transcript levels in pectoral muscle of the Myo-CELFΔ-370 line fall between levels measured in the hearts of the higher-expressing MHC-CELFΔ-10 and lower-expressing MHC-CELFΔ-574 lines (data not shown), so the lack of response in Myo-CELFΔ muscle is likely not due to insufficient transgene expression. It must be cautioned, however, that the level of residual CELF activity in the presence of the NLSCELFΔ protein cannot be directly measured, preventing a true comparison of the extent of repression of CELF activity in Myo-CELFΔ versus MHC-CELFΔ tissues. The possibility that NLSCELFΔ is less effective at inhibiting CELF activity in skeletal muscle than in heart muscle cells cannot be ruled out.

Other explanations for the weak or absent response of some targets are possible. For example, the lack of effect on some transcripts in Myo-CELFΔ muscle may simply reflect the developmental timing of when CELF proteins act on these targets. For example, in the heart Mfn2 exon 3 is partially skipped at embryonic stages when CELF1 and CELF2 protein levels are high, but is nearly 100% included in adult after CELF protein expression has decreased [4]. Over-expression of CELF1 is sufficient to recapitulate the embryonic splicing pattern of Mfn2 in the adult heart [4], but repression of CELF activity would not be expected to have any effect if the levels of CELF proteins in adult tissues are already below the threshold required to influence splicing of this exon. Targets such as Itgb1 exon D and Mef2A exon 16, however, are affected by NLSCELFΔ-mediated repression of CELF activity in the heart even at 24 weeks of age [22]. This suggests they should remain responsive in adult skeletal muscle, yet Myo-CELFΔ mice exhibit wild type splicing patterns for these transcripts.

Whatever the reason, it is nonetheless striking that the modest alternative splicing changes observed in Myo-CELFΔ mice were sufficient to induce changes in muscle organization, fiber size, and fiber subtype. Individual muscle fibers and muscle fascicles appear more compacted in Myo-CELFΔ muscle (Figure 3A). Although the functional consequences of reduced endomysial and perimysial spacing is not clear, connective tissue in the perimysium has been suggested to play a role in transmitting lateral contractile movements [26]. The extent of interstitial spacing in muscle appears to correlate with the level of CELF activity, as expansion and fibrosis of the muscle interstitia are observed in mouse models that over-express CELF1 in skeletal muscle [18], [20]. Large variations in muscle fiber size and an increase in the proportion of slow twitch muscle are observed in both Myo-CELFΔ mice (Figures 3B-F and 4) and mice over-expressing CELF1 [18], [20]. These results indicate that either too much or too little CELF activity will lead to the dysregulation of muscle fiber size and muscle fiber subtype. Notably, all three of these salient features affected in the Myo-CELFΔ phenotype – interstitial spacing, fiber size variability, and fiber type composition – are features that are affected in skeletal muscles from DM1 patients and mouse models of DM1 that express expanded CUG repeat RNAs [16], [27]–[29]. In addition to up-regulation of CELF1, sequestration and subsequent loss of function of muscleblind-like 1 (MBNL1) has been implicated in DM1 pathogenesis [30]. Notably, however, none of these features have been reported in MBNL1 knockout mice, which do exhibit other hallmarks of the disease such as centralized nuclei and split fibers [31]. Together, these results suggest that inappropriate CELF-mediated alternative splicing regulation may be largely responsible for these characteristics of DM1 muscle.

Roles for CELF proteins in normal skeletal muscle organization and composition have been suggested by previous studies in other animal models. The full complement of CELF family members arose from paralogous gene duplications in vertebrates, so invertebrate species possess only a single ortholog of CELF1/CELF2 [32]. RNAi-mediated inactivation of *etr-1*, the *Caenorhabditis elegans* homolog of CELF1/CELF2, causes embryonic lethality, accompanied by defects in muscle assembly, contractility, and attachment [33]. In vertebrates, repression of the *Xenopus* ortholog of CELF1 by either antisense knockdown or neutralizing antibodies in early frog embryos leads to defects in segmentation of the somites, which give rise to skeletal muscle [34]. In both *C. elegans* and *Xenopus*, early loss of CELF activity led to disorganization of muscle, but not perturbation of muscle differentiation *per se*. These results are consistent with our data that suggest that CELF activity is required for muscle organization, but not overall muscle mass. CELF1 knockout mice exhibit growth retardation, but a specific muscle phenotype has not been reported [35]. Early placentation defects in these mice may make it difficult to distinguish between specific changes in muscle mass and a general failure to thrive. CELF2 null mice have not yet been reported. In each of the worm, frog, and mouse knockdown/knockout models, the phenotype is more severe than that of the Myo-CELFΔ mice, which appear overtly healthy. This may be due to the difference between modulating total CELF protein levels and specific repression of CELF splicing activity. Both CELF1 and CELF2 are expressed in the cytoplasm as well as the nucleus in heart and skeletal muscle [4], [6], [36], and have been implicated in the regulation of cytoplasmic processes as well. For example, loss of CELF1 in *Xenopus* embryos has been shown to impair deadenylation [37], [38], and CELF1 has been implicated in translational regulation during muscle maturation in mice [39]. Therefore the contributions of disrupted cytoplasmic CELF activity and disrupted CELF-mediated alternative splicing to the phenotype may be difficult to separate in these models. Although the possibility of indirect effects on the cytoplasmic functions of CELF1 and CELF2 cannot be absolutely eliminated, the restriction of the dominant negative protein to the nucleus means the Myo-CELFΔ phenotype can most likely be attributed directly to changes in CELF-mediated alternative splicing.

## Materials and Methods

### Ethics statement

This study was conducted in strict accordance with the recommendations of the American Veterinary Medical Association and under the approval of the Institutional Animal Care and Use Committees of the Cleveland Clinic (Protocol numbers: ARC 07926 and ARC 08612) and Cleveland Veterans Affairs Medical Center (Protocol number: 06-15-EvL-MS1). All efforts were made to minimize pain and distress during animal husbandry, experimental assessments, and euthanasia.

### Generation of Myo-CELFΔ mice

The nuclear-restricted dominant negative CELF protein, NLSCELFΔ, was previously described [21]. The skeletal muscle-specific dominant negative transgene, Myo-CELFΔ, was created by subcloning the NLSCELFΔ open reading frame downstream of mouse myogenin 5′ flanking genomic sequence (provided by William Klein, University of Texas M.D. Anderson Cancer Center, Houston, TX) containing an ∼1.5 kb myogenin promoter, first intron, and 5′ end of exon 2, but in which the myogenin exon 1 was replaced by an exon from the 5′ untranslated region of β-globin; this promoter construct drives efficient expression of a green fluorescent protein reporter in mouse C2C12 myoblast cells that have been differentiated into myotubes following transient transfection (data not shown). The 3′ untranslated region of bovine growth hormone was amplified from the pcDNA3.1+ vector (Invitrogen) and subcloned downstream of the NLSCELFΔ open reading frame. The expression cassette was excised with XhoI and XbaI and microinjected into the pronuclei of fertilized B6SJL hybrid oocytes in the Case Transgenic and Targeting Facility (Case Western Reserve University, Cleveland, OH). Genotyping was performed by PCR amplification of mouse tails lysed in DirectPCR Lysis Reagent (Viagen Biotech, Inc.). A 410 bp Myo-CELFΔ transgene product (primers CCTAGAAGCTAGGAAAGACC and GTGGGAGGAGTACTTCACAAAG) and a 550 bp actin internal control product (primers GATGTGCTCCAGGCTAAAGTT and AGAAACGGAATGTTGTGGAGT) were amplified in the same reaction using an annealing temperature of 55°C and 30 cycles of amplification and resolved by agarose gel electrophoresis.

### Western blot analysis

Muscle tissue was homogenized in protein loading buffer (0.64 M Tris HCl, pH 6.8, 10% glycerol, 2% SDS, 5% b-mercaptoethanol), total protein samples were quantitated, resolved by polyacrylamide gel electrophoresis, transferred, and probed as previously described [5] using a monoclonal antibody against the N-terminal polyhistidine region or Xpress epitope tag (Invitrogen). Equivalent loading was confirmed by Ponceau S staining.

### Semi-quantitative RT-PCR

RT-PCR was performed as previously described [21], using conditions optimized for each primer set for amplification in the linear range with 250 ng total RNA: NLSCELFΔ primers GTATGGCTAGCATGACTGGTG and GCGTGGGAGGAGTACTTCAC, 62°C annealing temperature, and 24 cycles of amplification; 18S primers GGTAACCCGTTGAACCCCATTC and GGACCTCACTAAACCATCCAATCG, 56.7°C annealing temperature, and 8 cycles of amplification. Samples being compared were run in parallel with both primer sets, and NLSCELFΔ levels were normalized against 18S levels.

### Alternative splicing

Total RNA was extracted from hearts of transgenic and wild type littermates and subjected to single-tube RT-PCR analysis as previously described [21]. Mef2A, Itgb1, and Bin1 primers and PCR conditions are the same as used for MHC-CELFΔ mice [21]. Additional primer sets and reaction conditions are taken from Kalsotra and colleagues [4], with the exception of those for Nrap exon 12, which are from Lin and colleagues [25].

### Histology and fiber type analysis

Limbs of wild type and transgenic sex-matched littermates were transected and fixed overnight by immersion in 10% neutrally buffered formalin, embedded in paraffin, sectioned, and stained with hematoxilin and eosin. Interstitial area and fiber area were quantified using ImagePro Plus software (Media Cybernetics). For fiber type analysis, mice were anesthetized with an intraperitoneal injection of rodent anesthetic cocktail (ketamine, xylazine, and acepromazine), soleus muscles were surgically removed, embedded in tragacanth, rapidly frozen in isopentane cooled with liquid nitrogen, and sectioned on a cryostat. Sections were preincubated at room temperature in alkaline stock solution (75 mM glycine, 50 mM CaCl_2_, and 75 mM NaCl, pH 9.4) adjusted to pH 10.3 with NaOH for 15 min and rinsed three times with distilled water. Sections were then incubated in ATP solution (2.5 mM ATP in alkaline stock solution, pH 9.4) for 45 min at 37°C, and rinsed three times with distilled water. Sections were then serially incubated in 1% CaCl_2_ for 3 min, 2% CoCl_2_ for 3 min, and 1% NH_4_SH for 1 min, rinsing three times with distilled water after each. Sections were allowed to air dry and were mounted in Permount (Fisher Scientific). The number of dark and light fibers were counted using ImagePro Plus software. The samples sizes that are indicated are the number of individual mice in each group; replicate sections were independently stained and counted for each individual to ensure that observed differences are not due to variability in stain intensity between slides.

### *In vivo* grip strength assessments

Grip strength tests were performed in the Lerner Research Institute Rodent Behavioral Core using a digital grip strength meter (Columbus Instruments, Columbus, OH). To measure forelimb grip strength, each mouse was gently pulled away from the meter in a horizontal plane after it had gripped the bar, and the force applied to the bar immediately before the mouse released its grip was recorded. To measure hind limb grip strength, each mouse faced away from the meter with its forelimbs resting on the investigator's hand, and was pulled gently toward the meter in a horizontal plane. All mice underwent a training session the day prior to each session in which measurements were recorded. Animals completed 10 consecutive trials per session, and the measurements were averaged. Total body weight was measured at the end of each session. Grip strength measurements were made on the same individuals over a time course of 4, 8, 12, 16, 20, and 24 weeks of age and values were normalized to body weight.

### *Ex vivo* isometric muscle force and contractility measurements

For *ex vivo* isometric contractility assessments, mice were anesthetized and the diaphragm, EDL, and soleus muscles along with their tendons were surgically removed and kept in an aerated bath of physiological Krebs solution. The diaphragm was cut into strips, while the EDL and soleus were left intact. Each muscle or muscle strip was mounted vertically in a double-jacketed bath of aerated (95% O_2_/5% CO_2_) physiological solution (135 mM NaCl, 5 mM KCl, 2.5 mM CaCl_2_, 1 mM MgSO_4_, 1 mM NaH_2_PO_4_, 15 mM NaHCO_3_, and 11 mM glucose, pH adjusted to 7.35–7.45) at 37°C. Supramaximal voltages with a pulse width of 1 ms were delivered to muscles by a pair of platinum electrodes placed in parallel. The length of each muscle was adjusted to maximize twitch force. Following equilibration of the muscle, several baseline twitches were recorded. Muscles were subjected to an intermittent stimulation protocol in which a 333 ms stimulus train per minute was administered over a range of 1 to 120 Hz. Intermittent fatigue stimulation was used to test fatigue resistance for 300 sec at a frequency of either 25 or 50 Hz for a 333 ms train every second. Contractile performance was assessed by measuring peak force, contraction time (the time from the onset of force production to the top of the first peak of contraction), and half relaxation time (the time required for force to decrease 50% from the peak value at the end of stimulation).

### Statistics

Data are reported as mean ± standard error of the mean unless otherwise noted.

Contractility data were analyzed with repeated measures of analysis of variance followed by the Newman-Keuls test in the event of significance by analysis of variance. Comparisons between means for other data were performed via t-test assuming unequal variances using Microsoft Excel software. Comparisons of variances were performed via f-test using Microsoft Excel software. Differences were considered statistically significant when P≤0.05.

## Supporting Information

Figure S1 **Myo-CELFΔ transgenic mice exhibit normal body weight.** Body weights of Myo-CELFΔ-370 and wild type littermates from several litters were measured over a time course of 8, 12, 16, 20, and 24 weeks, and means for each group were determined. Males and females were assessed independently due to sexual dimorphism. Mean Myo-CELFΔ-370 body weights did not significantly differ from those of sex-matched wild type mice at any age.
(TIF)

Figure S2 **Muscle fiber number is not affected in Myo-CELFΔ mice.** Muscle fibers were counted in regions of transverse cross-sections of hind limbs using ImagePro Plus software (Media Cybernetics). Three different muscle groups were counted in each of two sex- and age-matched Myo-CELFΔ-370 and wild type littermate pairs, and mean values for each muscle group were compared. Muscle groups were chosen from regions where interstitial spaces were sufficiently evident in the Myo-CELFΔ-370 sections to allow for unambiguous identification of the corresponding muscle group in wild type sections.
(TIF)

Figure S3 **Muscle fatigue is not affected in skeletal muscles from Myo-CELFΔ mice.** Fatigue was assessed *ex vivo* by intermittent fatigue stimulation at 25 or 50 Hz in extensor digitorum longus (EDL) (**A**), soleus (**B**), and diaphragm (**C**) muscles isolated from sex- and age-matched wild type and Myo-CELFΔ-370 mice. No significant differences were found.
(TIF)

Figure S4 **Contraction time is not affected in skeletal muscles from Myo-CELFΔ mice.** The time from the onset of force production to the top of the first peak of contraction was measured *ex vivo* in EDL (**A**), soleus (**B**), and diaphragm (**C**) muscles isolated from sex- and age-matched wild type and Myo-CELFΔ-370 mice at 25 and 50 Hz. No significant differences were found.
(TIF)

Figure S5 **Half relaxation time is largely unaffected in skeletal muscles from Myo-CELFΔ mice.** The half relaxation time, calculated as the time required for force to decrease 50% from the peak value at the end of stimulation, was measured *ex vivo* in EDL (**A**), soleus (**B**), and diaphragm (**C**) muscles isolated from sex- and age-matched wild type and Myo-CELFΔ-370 mice at 25 and 50 Hz. No significant differences were found for EDL or soleus. A small but significant increase in half relaxation time was observed for the ends of the stimulation train in diaphragm.
(TIF)
