# Supplementary figures and images for: Expression of a Dominant Negative CELF Protein In Vivo Leads to Altered Muscle Organization, Fiber Size, and Subtype
Source: PLoS One. 2011 Apr 26;6(4):e19274. doi: 10.1371/journal.pone.0019274 (PMC3082560; doi:10.1371/journal.pone.0019274)

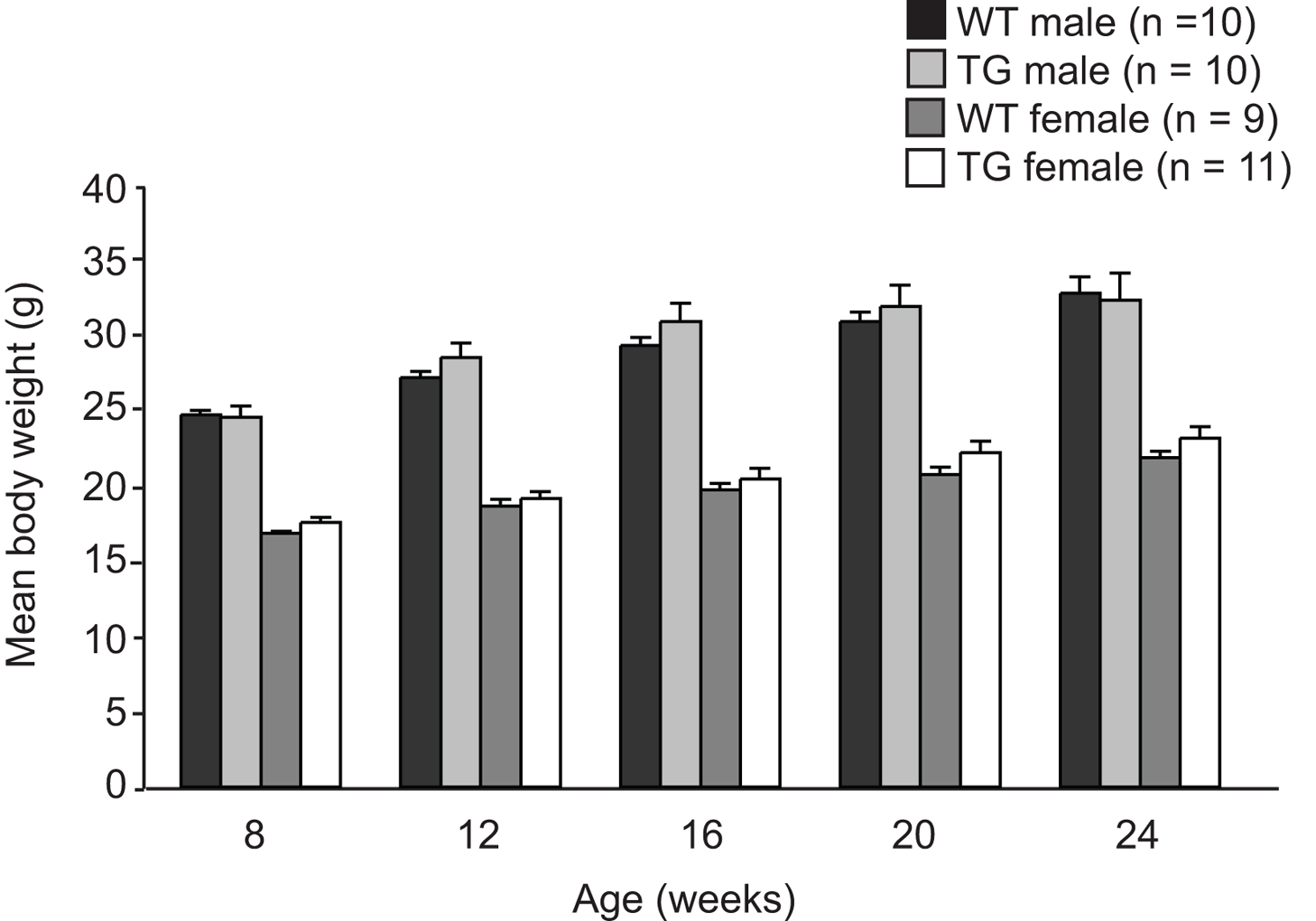

Supplement: Figure S1 — Myo-CELFΔ transgenic mice exhibit normal body weight. Body weights of Myo-CELFΔ-370 and wild type littermates from several litters were measured over a time course of 8, 12, 16, 20, and 24 weeks, and means for each group were determined. Males and females were assessed independently due to sexual dimorphism. Mean Myo-CELFΔ-370 body weights did not significantly differ from those of sex-matched wild type mice at any age. (TIF) [file pone.0019274.s001.tif]

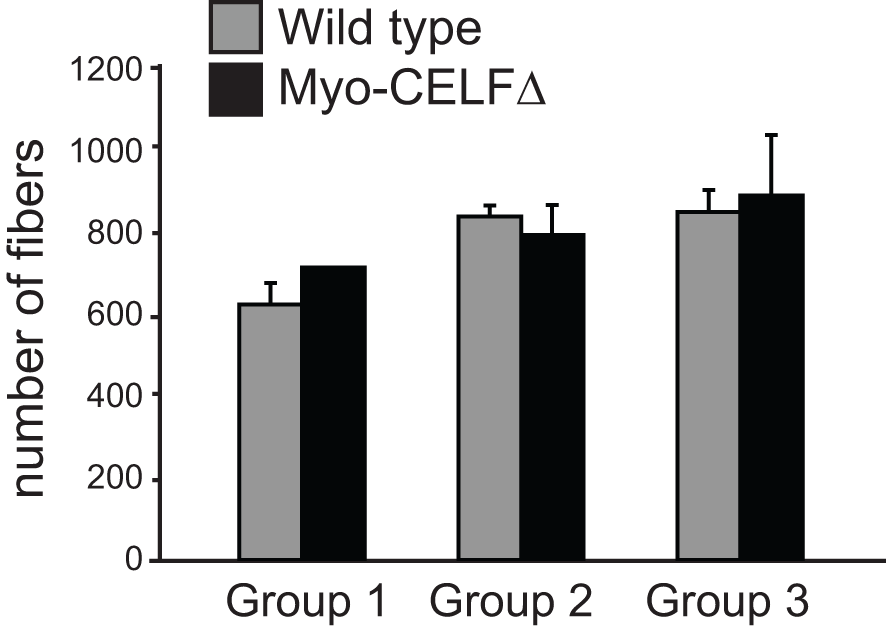

Supplement: Figure S2 — Muscle fiber number is not affected in Myo-CELFΔ mice. Muscle fibers were counted in regions of transverse cross-sections of hind limbs using ImagePro Plus software (Media Cybernetics). Three different muscle groups were counted in each of two sex- and age-matched Myo-CELFΔ-370 and wild type littermate pairs, and mean values for each muscle group were compared. Muscle groups were chosen from regions where interstitial spaces were sufficiently evident in the Myo-CELFΔ-370 sections to allow for unambiguous identification of the corresponding muscle group in wild type sections. (TIF) [file pone.0019274.s002.tif]

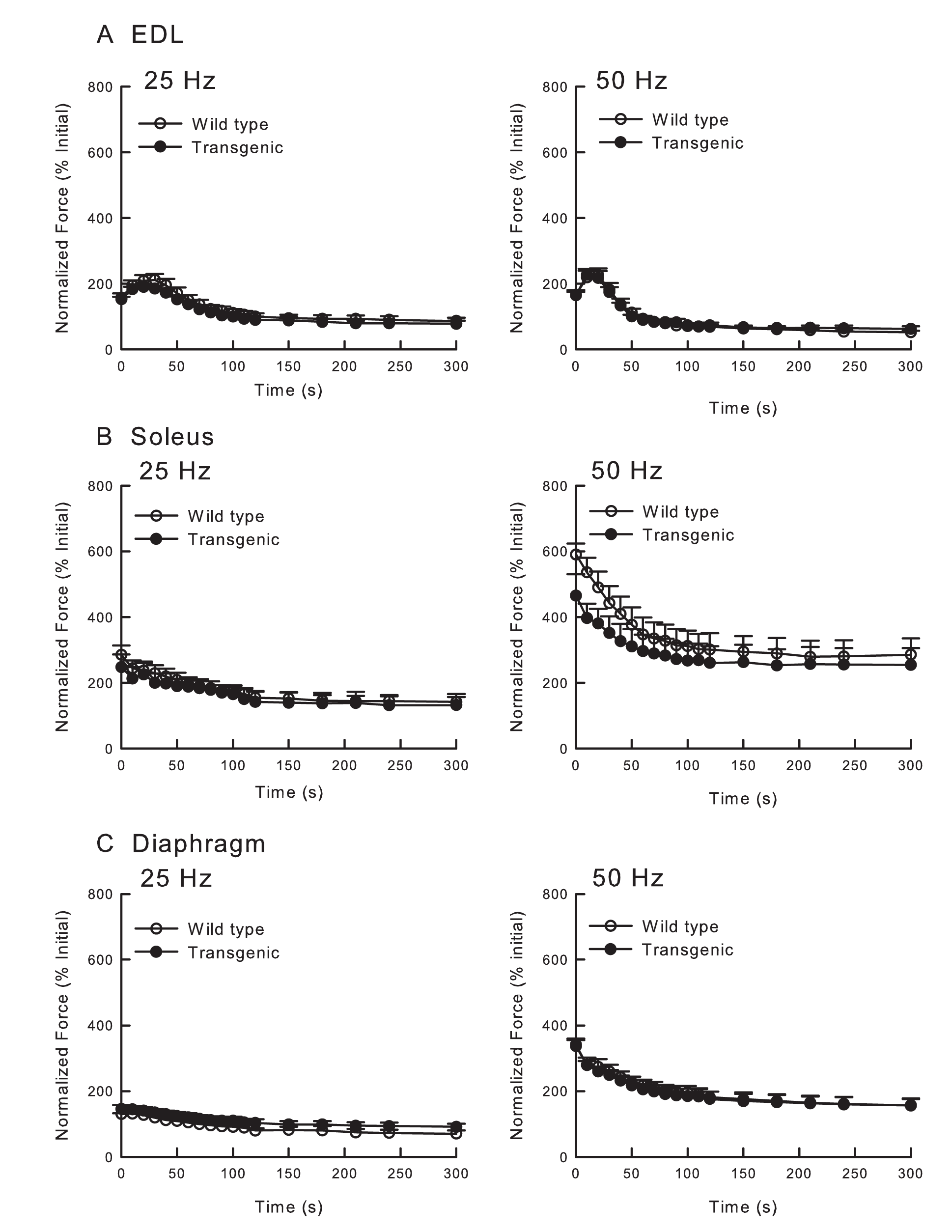

Supplement: Figure S3 — Muscle fatigue is not affected in skeletal muscles from Myo-CELFΔ mice. Fatigue was assessed ex vivo by intermittent fatigue stimulation at 25 or 50 Hz in extensor digitorum longus (EDL) (A), soleus (B), and diaphragm (C) muscles isolated from sex- and age-matched wild type and Myo-CELFΔ-370 mice. No significant differences were found. (TIF) [file pone.0019274.s003.tif]

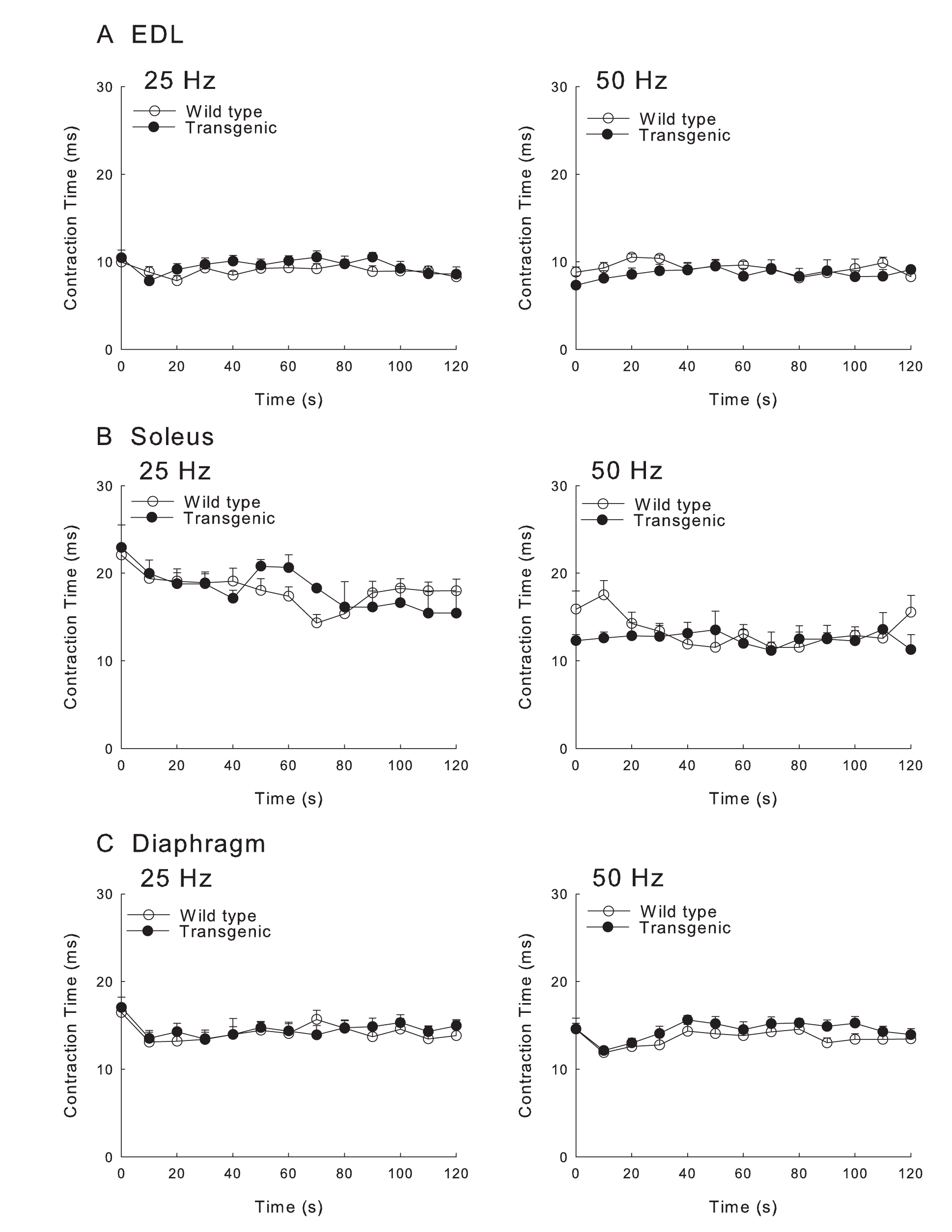

Supplement: Figure S4 — Contraction time is not affected in skeletal muscles from Myo-CELFΔ mice. The time from the onset of force production to the top of the first peak of contraction was measured ex vivo in EDL (A), soleus (B), and diaphragm (C) muscles isolated from sex- and age-matched wild type and Myo-CELFΔ-370 mice at 25 and 50 Hz. No significant differences were found. (TIF) [file pone.0019274.s004.tif]

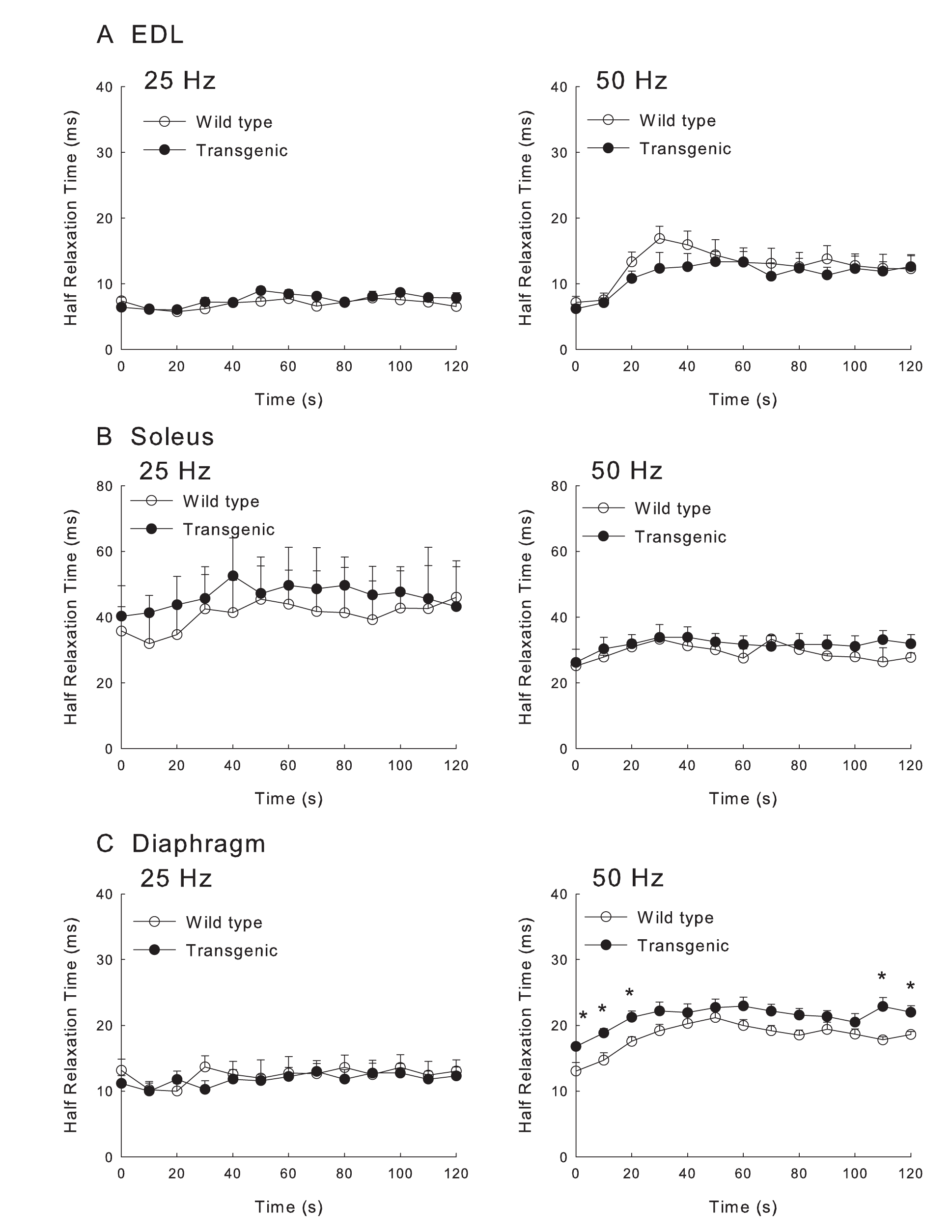

Supplement: Figure S5 — Half relaxation time is largely unaffected in skeletal muscles from Myo-CELFΔ mice. The half relaxation time, calculated as the time required for force to decrease 50% from the peak value at the end of stimulation, was measured ex vivo in EDL (A), soleus (B), and diaphragm (C) muscles isolated from sex- and age-matched wild type and Myo-CELFΔ-370 mice at 25 and 50 Hz. No significant differences were found for EDL or soleus. A small but significant increase in half relaxation time was observed for the ends of the stimulation train in diaphragm. (TIF) [file pone.0019274.s005.tif]
